# Development and internal validation of a parsimonious logistic regression model integrating multiparametric clinical indicators for predicting prostate cancer in the PI-RADS 3 cohort

**DOI:** 10.3389/fonc.2026.1883622

**Published:** 2026-07-22

**Authors:** Yongheng Zhou, Meikai Zhu, Yang Zheng, Wenfu Wang, Yaofeng Zhu

**Affiliations:** 1 Department of Urology, Qilu Hospital of Shandong University, Jinan, China; 2 University of Health and Rehabilitation Sciences, Qingdao, Shandong, China

**Keywords:** diagnosis, machine learning, multiparametric clinical indicators, PI-RADS 3, prostate cancer

## Abstract

**Objective:**

To develop and internally validate a parsimonious logistic regression model for clinically significant prostate cancer (CSPCa) specifically in patients with PI-RADS 3 lesions, integrating multiparametric clinical indicators to guide biopsy decision-making.

**Methods:**

We retrospectively enrolled 193 patients with PI-RADS 3 lesions who underwent mpMRI and prostate biopsy. A nested five-fold cross-validation framework was used, with LASSO regression performed independently in each training fold to select predictors from nine clinical variables. Four modeling approaches were compared, and logistic regression was chosen as the final model based on performance, calibration, and interpretability. Model discrimination, calibration, and clinical utility were evaluated internally.

**Results:**

LASSO selected age, PHI, and PHID as core predictors. Logistic regression achieved the highest cross-validated AUROC (0.840, 95% CI: 0.754–0.916) with acceptable calibration (slope 0.917, intercept −0.122). The model outperformed PSA and PSAD alone, and showed numerically higher discrimination than PHI or PHID alone. However, the incremental AUROC gain over PHI or PHID alone did not reach statistical significance. Exploratory risk stratification identified low-, intermediate-, and high-risk groups with CSPCa detection rates of 8.1%, 38.5%, and 83.3%, respectively.

**Conclusion:**

A three-variable logistic model incorporating age, PHI, and PHID demonstrated stable internal performance for predicting CSPCa in PI-RADS 3 patients. External validation is needed before clinical implementation.

## 1 Introduction

Prostate cancer (PCa) is the second most frequently diagnosed malignancy and the fifth leading cause of cancer death among men worldwide (1). Multiparametric MRI (mpMRI) has become central to prostate cancer diagnosis, with the Prostate Imaging-Reporting and Data System (PI-RADS) now established as the standard framework for reporting and risk stratification (2, 3). Although PI-RADS 4 and 5 scores reliably indicate clinically significant prostate cancer (CSPCa) warranting targeted biopsy, and scores of 1–2 suggest negligible risk, managing PI-RADS 3 lesions remains a clinical challenge due to their indeterminate nature (4, 5).

PI-RADS 3 lesions are characterized by an intermediate or equivocal probability of CSPCa. Guidelines from major bodies, such as the European Association of Urology (EAU) and the National Comprehensive Cancer Network (NCCN), diverge on management strategies for this cohort, ranging from active surveillance with repeat MRI to systematic or targeted biopsy (6, 7). This lack of consensus creates a clinical dilemma: many patients undergo unnecessary biopsies—exposing them to risks like infection, bleeding, and anxiety—while others may harbor missed CSPCa due to false-negative imaging (8). Indeed, reported detection rates for CSPCa in PI-RADS 3 lesions vary widely (10%–30%), underscoring the heterogeneity of this group and the critical need for more precise diagnostic tools (9–11).

Traditional biomarkers like PSA and its derivatives (e.g., free PSA, PSA density) have long been used to refine risk assessment, yet they often lack sufficient discriminative power when used in isolation (12). Recently, novel markers such as the Prostate Health Index (PHI) and PHI density (PHID) have shown promise in improving diagnostic accuracy (13, 14). Despite this potential, integrating these multiparametric indicators into a unified predictive framework using advanced analytical techniques remains underexplored, particularly for the PI-RADS 3 subpopulation.

Machine learning (ML) has become a transformative tool in medical informatics, uniquely capable of capturing complex, non-linear relationships within high-dimensional data that often elude traditional statistical methods (15, 16). While several studies have applied ML algorithms to predict PCa, most have focused on general populations or aggregated all PI-RADS categories. This approach risks obscuring the specific predictive value for the equivocal PI-RADS 3 cohort (17).

In this study, we compared four modeling approaches and ultimately developed an internally validated, parsimonious logistic regression model integrating multiparametric clinical indicators to predict CSPCa specifically in patients with PI-RADS 3 lesions. We evaluated the performance of four distinct approaches: logistic regression (LR), random forest (RF), extreme gradient boosting (XGBoost), and support vector machine (SVM). Furthermore, we constructed a clinically applicable risk stratification system designed to guide personalized biopsy decisions, ultimately optimizing the trade-off between cancer detection and the avoidance of unnecessary procedures.

## 2 Materials and methods

### 2.1 Study design and participants

This retrospective study included consecutive male patients with PI-RADS 3 lesions who underwent prostate mpMRI followed by prostate biopsy at Qilu Hospital of Shandong University from September 2020 to June 2025. All patients underwent MRI-targeted biopsy using software-based fusion systems that coregistered prior mpMRI lesions with real-time transrectal ultrasound, followed by systematic 12-core biopsy. Eligible patients were aged 40 years or older and had available clinical, laboratory, imaging and pathological data. Patients were excluded if they had a previous diagnosis of prostate cancer, prior prostate surgery, use of 5-alpha-reductase inhibitors within 6 months, acute prostatitis or urinary tract infection within 1 month, or incomplete data required for model development.

The study was approved by the Institutional Review Board of Qilu Hospital of Shandong University (KYLL-202011-095-2). The requirement for informed consent was waived because of the retrospective design.

### 2.2 Clinical variables and outcome definition

Clinical and laboratory variables were extracted from the medical records. Candidate predictors included age, total prostate-specific antigen (PSA), free PSA (fPSA), the free-to-total PSA ratio (F/T), [-2]proPSA (p2PSA), prostate volume (PV), PSA density (PSAD), Prostate Health Index (PHI) and Prostate Health Index density (PHID). Blood samples were obtained before biopsy. PSAD was calculated as total PSA divided by PV. PHI was calculated as (p2PSA/fPSA) x square root of total PSA, and PHID was calculated as PHI divided by PV. Prostate volume was measured on mpMRI using the ellipsoid formula.

All mpMRI examinations were assigned PI-RADS categories according to PI-RADS version 2.1 (3). The pathological reference standard was prostate biopsy. The primary endpoint was clinically significant prostate cancer (CSPCa), defined as Gleason score ≥3 + 4, corresponding to Grade Group ≥2. Non-CSPCa included benign pathology, prostatitis, high-grade prostatic intraepithelial neoplasia and Gleason score 3 + 3 prostate cancer.

### 2.3 Feature selection and collinearity assessment

Feature selection for the primary internal validation analysis was fully embedded within a nested resampling framework to eliminate data leakage. In each outer training fold of the 5-fold cross-validation, all nine candidate variables (age, PSA, fPSA, F/T, p2PSA, PV, PSAD, PHI and PHID) entered an L1-penalized (LASSO) logistic regression. The penalty parameter λ was selected by an inner 5-fold cross-validation using the 1-standard-error rule. Only variables with non-zero coefficients at the fold-specific optimal λ were carried forward to model fitting within that outer fold.

For descriptive presentation of the final model, an additional LASSO fit was performed on the full cohort only after completion of all internal validation procedures. Tuning parameter selection in this descriptive fit used 10-fold stratified cross-validation based on cross-validated log loss, and variables retained at both lambda.min and lambda.1se were recorded (**Supplementary Figure 1**; **Supplementary Table 1**). Collinearity assessment was performed for the final full-cohort presentation model with the three retained predictors. Variance inflation factors (VIFs) and tolerance values were calculated. A VIF greater than 5 was considered evidence of relevant multicollinearity.

### 2.4 Model development and internal validation

All model evaluations were conducted using a stratified nested 5-fold cross-validation framework on the full cohort, with stratification based on CSPCa status to preserve event rates across folds. Four modeling approaches were compared: logistic regression, random forest, extreme gradient boosting (XGBoost) and support vector machine (SVM). Within each outer training fold, each model was fitted using only the predictors selected by the fold-specific inner LASSO procedure, ensuring that held-out test patients were never exposed to variable selection or model training. Logistic regression and SVM were fitted after standardization of predictors. Random forest used 500 trees with the square-root rule for the number of candidate variables at each split. XGBoost used 300 estimators, a maximum tree depth of 3, a learning rate of 0.05, subsampling of 0.90 and column subsampling of 0.90. The SVM used a radial basis function kernel with C = 1.0 and gamma set to scale.

All cross-validation procedures were run with shuffling and a fixed random seed for reproducibility. For each patient, the predicted probability used for all performance assessments was an out-of-fold prediction generated from a model that had neither been trained on that patient nor exposed to that patient’s outcome during feature selection. These out-of-fold predictions were used to calculate all performance metrics, including AUROC, Brier score, calibration intercept and slope, threshold-based sensitivity and specificity, decision curve analysis, and exploratory risk stratification. The final model was selected on the basis of discrimination, calibration and clinical interpretability. Logistic regression was retained as the final model because it showed the best overall performance and could be presented transparently as a nomogram.

After completion of all internal validation procedures, a final presentation logistic regression model was refitted on the full cohort using the three predictors retained in the descriptive full-cohort LASSO fit (age, PHI and PHID). This full-cohort model was used exclusively for nomogram construction and descriptive presentation of predictor effects. Its apparent in-sample performance was reported only as a secondary descriptive estimate, and all primary performance conclusions were based on the nested cross-validated out-of-fold predictions, supplemented by bootstrap optimism correction.

### 2.5 Performance metrics and calibration

Discrimination was assessed using the area under the receiver operating characteristic curve (AUROC). Bootstrap resampling with 1,000 iterations was used to estimate 95% confidence intervals for AUROC. Overall prediction error was assessed using the Brier score. The Brier score was not interpreted as a calibration-specific metric because it is affected by discrimination and outcome prevalence.

Calibration curves were generated from internally cross-validated out-of-fold predictions grouped into five predicted-risk quantiles; observed event rates were shown with binomial confidence intervals. Because these plots were based on internal validation only, they were interpreted cautiously and used as descriptive rather than definitive calibration evidence.

Sensitivity, specificity and confusion-matrix counts were calculated at a fixed predicted-probability threshold of 0.50. This threshold was used only to summarize binary classification performance and was not selected as an optimized clinical decision threshold. The number of false-negative CSPCa cases was reported to avoid overemphasizing high specificity without accounting for missed cancers.

For the final logistic regression model, bootstrap optimism correction was performed with 1,000 bootstrap resamples. The model was refitted in each bootstrap sample and tested in the original cohort to estimate optimism in AUROC, Brier score, calibration intercept and calibration slope.

### 2.6 Benchmarking against clinical predictors

To evaluate whether the multivariable model added information beyond clinically available markers, the final logistic model was compared with PHID alone, PSAD alone, PHI alone and PSA alone. Each single-predictor benchmark was fitted as a one-variable logistic model and evaluated using the same five-fold cross-validated out-of-fold prediction framework. The difference in AUROC between the final model and each benchmark was estimated by paired bootstrap resampling with 1,000 iterations.

### 2.7 Exploratory risk stratification and decision curve analysis

Exploratory risk strata were defined using cross-validated predicted probabilities: low risk, <0.20; intermediate risk, 0.20-0.70; and high risk, ≥0.70. These cutoffs were prespecified as exploratory probability bands for descriptive risk stratification and were not treated as validated clinical decision thresholds. CSPCa detection rates and 95% confidence intervals were reported for each group.

Decision curve analysis was performed using cross-validated predicted probabilities from the final logistic model. Net benefit was calculated across threshold probabilities from 0.01 to 0.80 and compared with treat-all and treat-none strategies. This analysis was considered exploratory because no external validation cohort was available.

### 2.8 Statistical analysis

Continuous variables were summarized as medians and interquartile ranges, and categorical variables were summarized as counts and percentages. Between-group comparisons were performed using the Mann-Whitney U test for continuous variables and the chi-square test or Fisher exact test for categorical variables, as appropriate. All statistical tests were two-sided, and P < 0.05 was considered statistically significant. Analyses were performed in Python using scikit-learn, XGBoost and statsmodels.

## 3 Results

### 3.1 Patient characteristics

A total of 193 patients with PI-RADS 3 lesions were included, of whom 41 had clinically significant prostate cancer (CSPCa). The age distribution differed between patients with and without CSPCa [median age, 66 years (IQR, 63-74) vs 66 years (IQR, 60-70); P = 0.038], despite identical median values. Several prostate cancer-related biomarkers differed between groups. p2PSA, PSAD, PHI and PHID were significantly higher in patients with CSPCa, whereas prostate volume was significantly lower (P = 0.001 for p2PSA and PSAD; P < 0.001 for PV, PHI and PHID). PSA and fPSA did not differ significantly between groups (**Table 1**).

**Table 1:** Baseline characteristics of patients.

Variable	Overall (N = 193)	Non-CSPCa (N = 152)	CSPCa (N = 41)	P-value
Age, median (IQR)	66.00 (61.00 - 70.00)	66.00 (60.00 - 70.00)	66.00 (63.00 - 74.00)	0.038
PSA, median (IQR)	8.66 (6.28 - 11.43)	8.22 (6.20 - 11.32)	9.48 (7.08 - 12.86)	0.142
fPSA, median (IQR)	1.30 (0.91 - 1.86)	1.32 (0.96 - 1.96)	1.14 (0.82 - 1.45)	0.421
F/T, median (IQR)	0.15 (0.11 - 0.20)	0.16 (0.12 - 0.21)	0.13 (0.10 - 0.16)	0.021
p2PSA, median (IQR)	17.38 (11.65 - 26.57)	16.16 (10.89 - 23.99)	26.57 (18.09 - 32.82)	0.001
PV, median (IQR)	53.58 (36.14 - 74.95)	60.56 (39.64 - 77.07)	36.14 (28.42 - 55.37)	<0.001
PSAD, median (IQR)	0.16 (0.11 - 0.24)	0.15 (0.11 - 0.21)	0.24 (0.16 - 0.30)	0.001
PHI, median (IQR)	42.29 (28.92 - 57.98)	38.04 (27.61 - 48.51)	71.63 (46.75 - 89.70)	<0.001
PHID, median (IQR)	0.72 (0.44 - 1.35)	0.63 (0.41 - 0.98)	1.88 (1.23 - 2.96)	<0.001

### 3.2 Feature selection and collinearity

In the nested cross-validation workflow, PHI and PHID were selected in all five outer training folds, whereas age was selected in three of five folds. The two dominant feature sets were PHI + PHID and age + PHI + PHID, indicating that the final three-variable model was directionally stable but that the contribution of age was modest. In the descriptive full-cohort analysis performed after completion of internal validation, LASSO logistic regression was used to summarize the final predictor set. At both lambda.min and lambda.1se, age, PHI and PHID retained non-zero coefficients, whereas PSA, fPSA, F/T, p2PSA, PV and PSAD were shrunk to zero (**Supplementary Figure 1**; **Supplementary Table 1**). Collinearity analysis of the selected predictors showed no substantial multicollinearity. The VIF values were 1.053 for age, 2.129 for PHI and 2.185 for PHID, all below the prespecified threshold of 5 (**Supplementary Table 2**).

### 3.3 Model comparison in full-cohort internal validation

**Figure 1:**
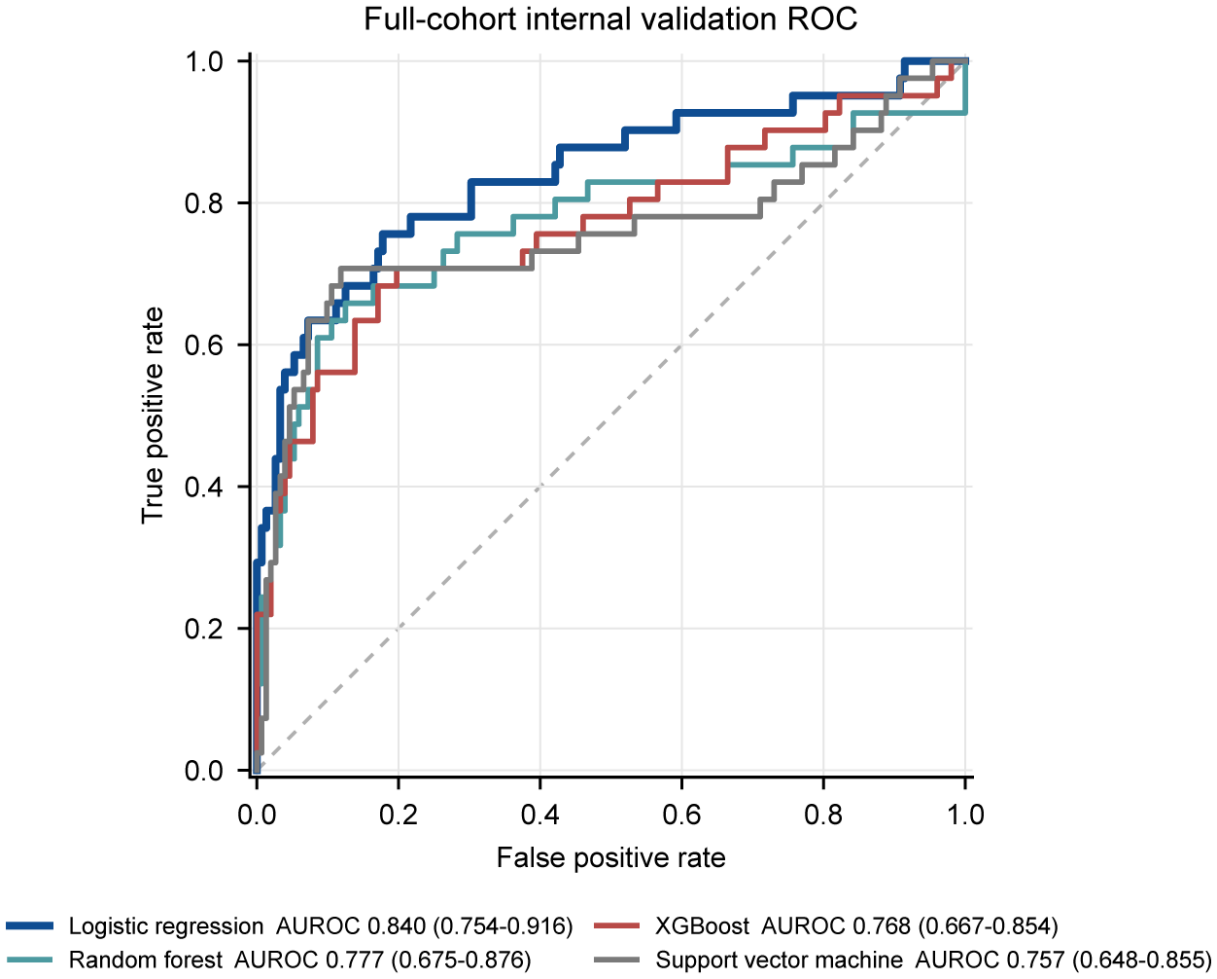
Receiver operating characteristic curves for four prediction models in full-cohort internal validation. Curves are generated based on 5-fold cross-validated out-of-fold predictions from 193 patients with PI-RADS 3 lesions (41 CSPCa events). Feature selection was performed independently within each training fold to avoid data leakage. AUROC values with 95% confidence intervals are presented for each algorithm: logistic regression (0.840, 0.754–0.916), random forest (0.777, 0.675–0.876), XGBoost (0.768, 0.667–0.854), and support vector machine (0.757, 0.648–0.855). The dashed diagonal line represents the reference of random prediction.

Four models were evaluated using fold-wise LASSO-selected predictors and five-fold cross-validated out-of-fold predictions. Logistic regression showed the highest AUROC among the four models (0.840, 95% CI: 0.754-0.916), followed by random forest (0.777, 95% CI: 0.675-0.876), XGBoost (0.768, 95% CI: 0.667-0.854) and support vector machine (0.757, 95% CI: 0.648-0.855; **Figure 1**; **Table 2**). The logistic model also had the lowest Brier score (0.105), indicating the lowest overall prediction error among the compared models. Calibration measures for logistic regression remained reasonably close to ideal, with a calibration intercept of -0.122 and a calibration slope of 0.917. At the 0.50 probability threshold, sensitivity values across the four models ranged from 0.463 to 0.537, while the logistic regression model achieved the highest specificity (0.967). The other three models exhibited poorer calibration, with calibration slopes ranging from 0.321 to 0.852 and intercepts deviating markedly from the ideal value of zero.

**Table 2:** Model performance in the full-cohort internal validation analysis.

Model	AUROC (95% CI)	Sensitivity (95% CI)	Specificity (95% CI)	Brier score	Calibration intercept	Calibration slope
Logistic regression	0.840 (0.754-0.916)	0.512 (0.365-0.657)	0.967 (0.925-0.986)	0.105	-0.122	0.917
Random forest	0.777 (0.675-0.876)	0.488 (0.343-0.635)	0.947 (0.900-0.973)	0.121	-0.683	0.321
XGBoost	0.768 (0.667-0.854)	0.463 (0.321-0.613)	0.947 (0.900-0.973)	0.128	-0.394	0.520
Support vector machine	0.757 (0.648-0.855)	0.537 (0.387-0.679)	0.947 (0.900-0.973)	0.115	-0.280	0.852

Performance estimates were derived from 5-fold cross-validated out-of-fold predictions in the full cohort, with feature selection embedded within each training fold by LASSO logistic regression. The final displayed predictor set for the logistic model was age, PHI and PHID. Classification metrics were calculated using a probability threshold of 0.50. AUROC 95% confidence intervals were estimated using 1,000 patient-level bootstrap resamples. AUROC, area under the receiver operating characteristic curve; CI, confidence interval; CSPCa, clinically significant prostate cancer; PHI, Prostate Health Index; PHID, PHI density; XGBoost, extreme gradient boosting.

### 3.4 Internal validation of the final logistic regression model

**Figure 2:**
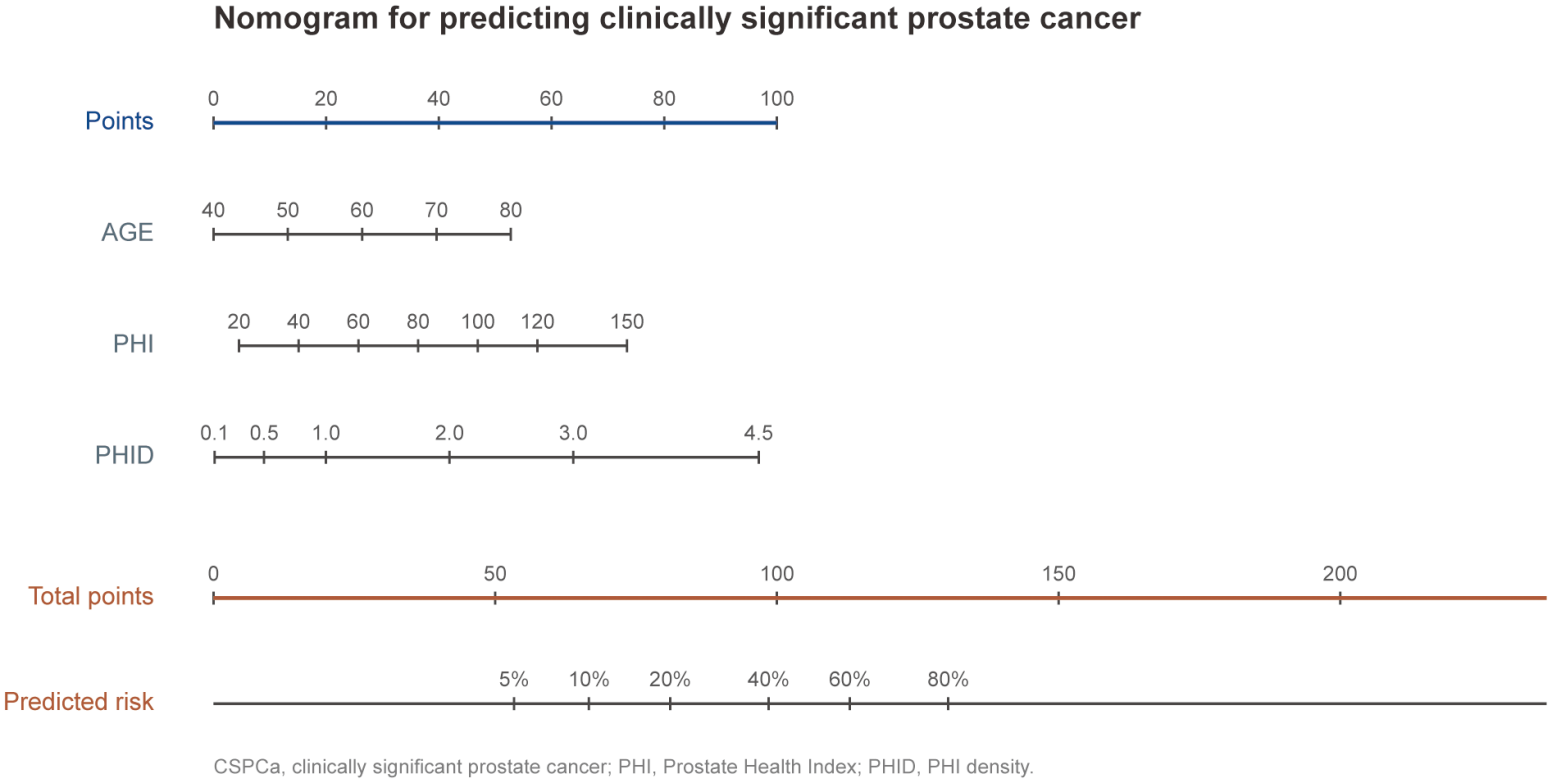
Nomogram for predicting clinically significant prostate cancer. The nomogram is constructed based on the final three-variable logistic regression model fitted in the full cohort, with age, PHI, and PHID as independent predictors. Points assigned to each variable are summed to the total points scale, which corresponds to the individual predicted probability of CSPCa. CSPCa, clinically significant prostate cancer; PHI, Prostate Health Index; PHID, PHI density.

**Figure 3:**
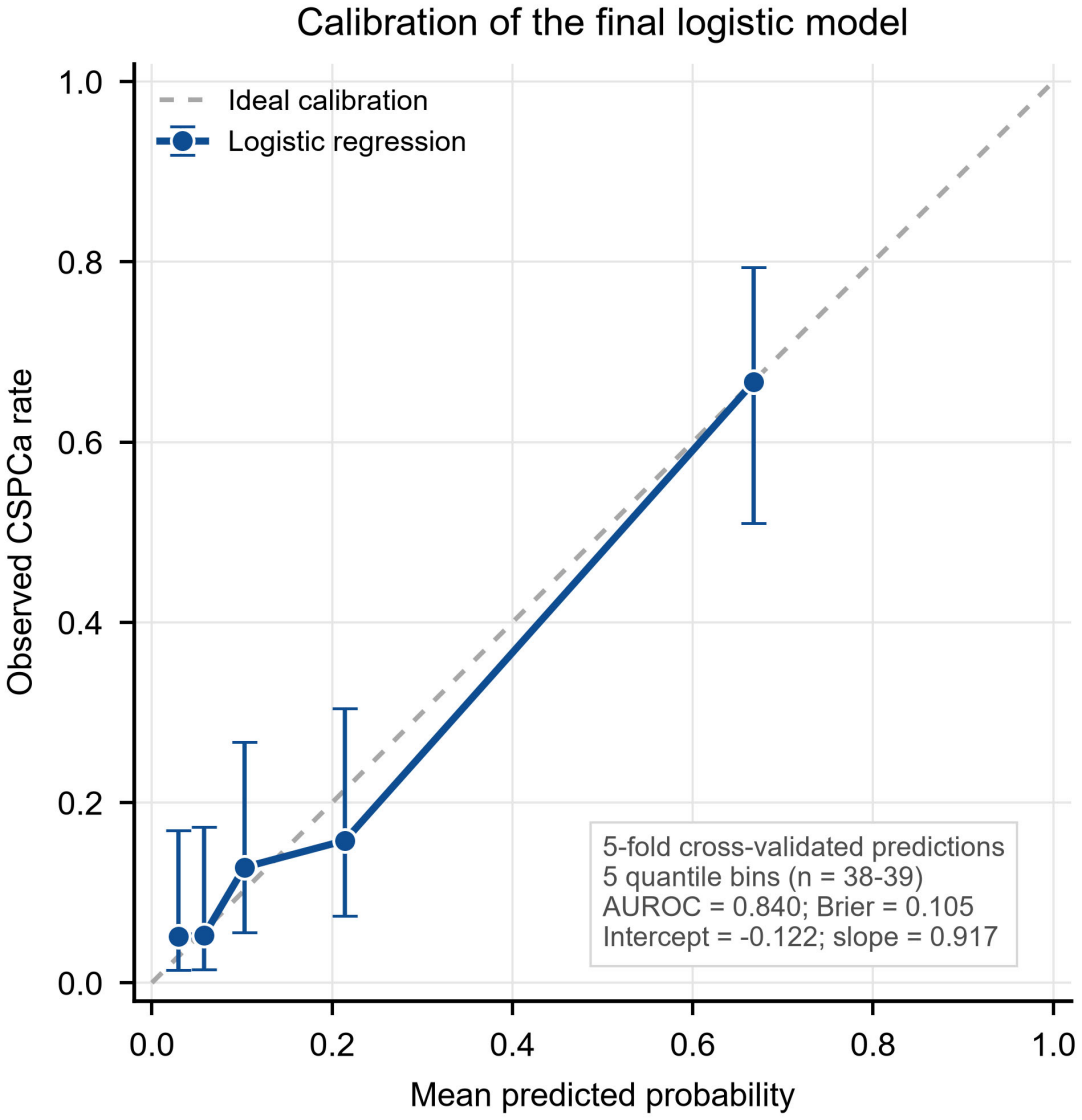
Calibration curve of the final logistic regression model. The curve is derived from 5-fold cross-validated out-of-fold predictions grouped into 5 quantile bins (38–39 patients per bin). Feature selection was performed independently within each training fold. The x-axis indicates the mean predicted probability of CSPCa, and the y-axis indicates the observed CSPCa rate. Error bars represent binomial 95% confidence intervals for observed event rates. The dashed gray line denotes ideal calibration (predicted probability equals observed rate). The model achieved an AUROC of 0.840, a Brier score of 0.105, a calibration intercept of −0.122, and a calibration slope of 0.917.

The final logistic regression model included age, PHI and PHID and was used to construct the nomogram (**Figure 2**). In five-fold cross-validation, the model achieved an AUROC of 0.840 (95% CI: 0.754-0.916), sensitivity of 0.512 (95% CI: 0.365-0.657) and specificity of 0.967 (95% CI: 0.925-0.986) at a fixed probability threshold of 0.50. At this threshold, the model identified 21 of 41 CSPCa cases and missed 20 CSPCa cases, corresponding to a false-negative proportion of 48.8% among patients with CSPCa. For clinical context, we also evaluated performance at the 0.20 probability threshold, which defines the boundary of the exploratory low-risk stratum. At this lower threshold, sensitivity increased to 0.732 (95% CI: 0.581–0.843) and specificity decreased to 0.822 (95% CI: 0.754–0.875), with 11 of 41 CSPCa cases missed (26.8% false-negative rate among CSPCa patients). Full threshold-based metrics are summarized in **Supplementary Table 3**. The threshold-based results were therefore used only as descriptive classification summaries rather than as a rule to defer biopsy. The bootstrap optimism-corrected AUROC was 0.862, with a Brier score of 0.097, calibration intercept of 0.019 and calibration slope of 1.013. The apparent full-cohort model had an AUROC of 0.867 (95% CI: 0.790-0.936), but this estimate was interpreted as optimistic because the same data were used for model fitting and evaluation (**Table 3**). The calibration curve based on internally cross-validated grouped predictions is shown in **Figure 3**.

**Table 3:** Full-cohort internal validation of the final logistic regression model.

Validation analysis	n/CSPCa events	AUROC (95% CI)	Brier score	Calibration intercept	Calibration slope	Sensitivity (95% CI)	Specificity (95% CI)
5-fold cross-validated out-of-fold predictions	193/41	0.840 (0.754-0.916)	0.105	-0.122	0.917	0.512 (0.365-0.657)	0.967 (0.925-0.986)
Bootstrap optimism-corrected model	193/41	0.862	0.097	0.019	1.013	NA	NA
Apparent full-cohort model	193/41	0.867 (0.790-0.936)	0.092	0.077	1.064	0.512 (0.365-0.657)	0.980 (0.944-0.993)

The final logistic regression model was presented using age, PHI, and PHID. Five-fold cross-validated estimates were derived from out-of-fold predicted probabilities in the full cohort, with LASSO feature selection repeated within each outer training fold. Bootstrap optimism-corrected estimates were obtained from 1,000 bootstrap resamples. The Brier score measures overall probabilistic prediction error rather than calibration alone. Calibration intercept and slope were estimated by regressing the observed outcome on the logit of the predicted probability. Classification metrics were calculated using a probability threshold of 0.50. AUROC, area under the receiver operating characteristic curve; CI, confidence interval; CSPCa, clinically significant prostate cancer; PHI, Prostate Health Index; PHID, PHI density.

### 3.5 Incremental value over single clinical predictors

To assess whether the multivariable model added information beyond available clinical predictors, we compared the final three-variable logistic model with PHID alone, PSAD alone, PHI alone and PSA alone using the same full-cohort five-fold cross-validated out-of-fold predictions (**Supplementary Table 4**). The final model achieved an AUROC of 0.840 (95% CI, 0.754-0.916), compared with 0.811 (95% CI, 0.718-0.894) for PHID alone, 0.824 (95% CI, 0.739-0.899) for PHI alone, 0.711 (95% CI, 0.621-0.798) for PSAD alone and 0.569 (95% CI, 0.463-0.672) for PSA alone. The AUROC gain was significant compared with PSAD alone (delta AUROC, 0.128; 95% CI, 0.035-0.225; P = 0.006) and PSA alone (delta AUROC, 0.269; 95% CI, 0.153-0.384; P < 0.001), but not compared with PHID alone (delta AUROC, 0.028; 95% CI, -0.026 to 0.079; P = 0.284) or PHI alone (delta AUROC, 0.015; 95% CI, -0.025 to 0.056; P = 0.422). The final model also showed the lowest Brier score among the evaluated approaches (0.105), with net benefits of 0.120 and 0.083 at threshold probabilities of 0.20 and 0.50, respectively.

### 3.6 Exploratory risk stratification

Using the internally cross-validated predicted probabilities, patients were stratified into exploratory low-risk (<0.20), intermediate-risk (0.20-0.70) and high-risk (>=0.70) groups. The low-risk group included 136 patients (70.5%), among whom 11 had CSPCa, corresponding to a detection rate of 8.1% (95% CI: 4.6-13.9). The intermediate-risk group included 39 patients (20.2%), with 15 CSPCa cases and a detection rate of 38.5% (95% CI: 24.9-54.1). The high-risk group included 18 patients (9.3%), with 15 CSPCa cases and a detection rate of 83.3% (95% CI: 60.8-94.2; **Table 4**).

**Table 4:** Exploratory risk stratification based on internally cross-validated predicted probabilities.

Risk category	Probability range	Patients, n (%)	CSPCa cases, n	CSPCa detection rate, % (95% CI)
Low risk	<0.20	136 (70.5)	11	8.1 (4.6-13.9)
Intermediate risk	0.20-0.70	39 (20.2)	15	38.5 (24.9-54.1)
High risk	≥0.70	18 (9.3)	15	83.3 (60.8-94.2)

Risk categories were based on 5-fold internally cross-validated predicted probabilities from the final logistic regression model, with feature selection embedded within each training fold and the displayed final predictors given as age, PHI, and PHID. Confidence intervals were calculated using the Wilson method. These thresholds are exploratory probability bands and should not be interpreted as externally validated clinical decision cutoffs. CSPCa, clinically significant prostate cancer; CI, confidence interval; PHI, Prostate Health Index; PHID, PHI density.

These probability bands were not treated as validated biopsy thresholds. Decision curve analysis was performed as an exploratory assessment of clinical utility (**Supplementary Figure 2**). The final model yielded higher net benefit than both the treat-all and treat-none strategies across most clinically relevant threshold probabilities (0.01–0.80), indicating favorable clinical utility.

## 4 Discussion

In this study, we developed an internally validated prediction model for CSPCa among patients with PI-RADS 3 lesions using a stratified nested 5-fold cross-validation framework, in which LASSO-based feature selection was performed independently within each outer training fold via inner cross-validation to fully avoid information leakage. LASSO regression, implemented via the coordinate descent algorithm for generalized linear models with convex penalties (18), selected age, PHI and PHID as the final predictors in the descriptive full-cohort fit. In the nested cross-validation procedure, PHI and PHID were selected in all five outer training folds, whereas age was retained in three of five folds, indicating stable core predictors with a modest contribution from age. Among the four evaluated algorithms, logistic regression showed the best overall balance of discrimination, calibration and parsimony. Consistent with previous findings comparing regularized logistic regression with other algorithms in prostate cancer cohorts, we observed that the logistic model delivered more stable performance with a smaller training-to-validation AUC drop, suggesting limited overfitting (19). Its cross-validated AUROC was 0.840, and its calibration slope was 0.917, suggesting acceptable calibration under the strict internal validation framework.

The selection of PHI and PHID is clinically plausible. PHI combines PSA isoforms into a single biomarker, while PHID adjusts PHI by prostate volume. Accumulating evidence shows that volume-adjusted biomarkers consistently achieve higher diagnostic accuracy than unadjusted markers for CSPCa detection, especially in patients with equivocal PI-RADS lesions (20). This may explain why PSAD, although different between CSPCa and non-CSPCa groups in the univariate comparison, was not retained after LASSO selection. In other words, PHID may capture part of the information carried by prostate density while preserving the contribution of PHI. Age remained in the model, but its VIF was low, indicating that it added information without introducing collinearity.

The added value of the model should be interpreted against clinically available comparators rather than against raw PSA alone. In this analysis, the final model performed clearly better than PSA and PSAD alone, and it also had numerically higher discrimination and a lower Brier score than PHI or PHID alone. The comparison with PHID is particularly important because PHID already combines PHI with prostate volume and is therefore a strong clinical benchmark. A large prospective European cohort demonstrated that PHID significantly improved diagnostic accuracy over PHI and PSAD, with an AUC of 0.835 versus 0.801 for PHI and 0.726 for PSAD, and decision curve analysis confirmed its superior net benefit across clinically meaningful threshold probabilities (21). Similarly, a prospective study in an Asian population confirmed that PHID outperformed PHI and tPSA in the PSA gray zone, supporting the utility of PHID as a robust clinical benchmark across different ethnic populations (22). The improvement over PHID did not reach statistical significance in the present cohort, which means that the model should not be presented as a definitive replacement for PHID. A more balanced interpretation is that adding age to PHI and PHID produced a small, internally validated improvement in overall prediction while preserving a simple and clinically interpretable structure. In contrast to prospective cohorts that systematically recorded digital rectal examination findings and family history and successfully performed external validation of their locally developed risk calculators (23), our retrospective dataset did not contain all variables required by established external risk calculators. Therefore, those calculators could not be evaluated directly. External validation against PHID and established risk calculators is still needed before clinical use.

The exploratory comparison across algorithms did not support a move toward a more complex ML model. A systematic review of head-to-head comparisons across multiple ML methods and logistic regression reported no consistent performance advantage for ML over logistic regression in clinical prediction tasks, with all methods achieving comparable discrimination (24). In line with this, XGBoost (0.768), random forest (0.777) and support vector machine (0.757) all produced lower AUROC values than logistic regression (0.840) in the same 5-fold nested cross-validation setting. However, it should be acknowledged that recent studies in similar patient populations have reported conflicting results. Özlü and colleagues developed ML models incorporating PSA-related variables, mpMRI findings, and hematologic parameters in biopsy candidates with PSA < 10 ng/mL, and found that XGBoost achieved an AUC of 0.97 with 94.74% sensitivity and 100% specificity, while random forest yielded an AUC of 0.89, with f/tPSA ratio, PI-RADS score 4, and platelet-to-lymphocyte ratio emerging as the most influential predictors (25). The discrepancy between their findings and ours may be attributable to differences in cohort composition: their study included patients across all PI-RADS categories with a PCa detection rate of 15.5%, whereas our cohort was restricted to PI-RADS 3 lesions, which represent a diagnostically more equivocal subgroup and may limit the discriminative ceiling of complex algorithms. Their calibration was also less favorable, especially for the tree-based models. From a methodological perspective, tree-based algorithms such as random forest and XGBoost have been reported to exhibit less favorable calibration, with higher Brier scores and calibration slopes deviating further from unity compared to logistic regression, which may undermine the reliability of risk estimates in clinical decision-making (26). Given the modest sample size, the inherent uncertainty of PI-RADS 3 lesions, and the need for interpretability in biopsy decision support, the logistic model was retained as the final model. This choice also allowed the model to be represented as a nomogram, which may be easier to review and apply in clinical settings.

The risk stratification analysis should be interpreted cautiously. The low-risk group had a lower CSPCa detection rate than the intermediate- and high-risk groups, but CSPCa was still present in 11 of 136 patients. At the fixed 0.50 probability threshold, the model also missed 20 of 41 CSPCa cases. Therefore, the model should not be used alone to rule out biopsy. Clinically, the fixed 0.50 threshold carries a notable risk of missed cancers because it prioritizes high specificity. To minimize false-negative cases, a lower probability threshold, such as the 0.20 cutoff used to define the low-risk stratum, can substantially improve sensitivity and reduce the number of missed CSPCa cases, though it will also increase the rate of unnecessary biopsies. Conversely, a higher threshold may be acceptable for patients with limited life expectancy or a strong preference to avoid biopsy. In practice, no single threshold fits all patients, and biopsy decisions should be individualized by combining model-derived risk with clinical context. PI-RADS 3 lesions represent an indeterminate imaging category with considerable overlap with benign conditions and substantial interobserver variability, and current clinical practice evidence recommends combining mpMRI findings with serum biomarkers to refine risk estimates rather than basing biopsy decisions on imaging alone (27, 28). A more appropriate interpretation is that the model may support shared decision-making by placing a PI-RADS 3 patient into a more individualized risk range. A Chinese multicenter study demonstrated that a model integrating PSAD and PI-RADS score can stratify patients into low-, intermediate-, and high-risk groups, providing individualized risk estimates that facilitate shared decision-making for patients with equivocal MRI findings (29). Similarly, combining MRI-derived parameters such as lesion size, DWI score, and location with serum biomarkers (PSAD and PHI) significantly improved risk stratification for PI-RADS 3 transition zone lesions, supporting the notion that biopsy decisions should not be based on imaging findings alone (28). For equivocal gray-zone populations, reliable calibration and clinical net benefit are often more clinically valuable than a marginal gain in discrimination, as they directly support more accurate individualized biopsy decision-making (30). The high specificity at the 0.50 threshold should be read together with the modest sensitivity and the corresponding missed-cancer burden. In exploratory stratification, patients in the low-risk group may be candidates for shared decision-making and closer follow-up, yet the model alone is insufficient to defer biopsy. Patients in the intermediate-risk group require individualized biopsy decisions integrated with full clinical context, while those in the high-risk group warrant stronger consideration of biopsy, pending further external validation.

This study has several limitations. First, all analyses were based on a single full cohort with internal validation. Although cross-validation and bootstrap correction reduce optimism, they do not replace external validation. As emphasized in recent ML studies in this field, external validation in diverse populations with larger patient numbers remains essential to confirm the generalizability of predictive models (25). Second, the number of CSPCa events was limited, resulting in an events-per-variable (EPV) ratio of approximately 13.7 for the final three-predictor model, which does not meet the commonly recommended empirical threshold of 20. This is an inherent data limitation of our single-center retrospective cohort and cannot be fully resolved by statistical adjustments. It reduces the precision of sensitivity and calibration estimates, and likely contributed to some predictor-level instability, as age was selected in three of the five outer folds rather than all five. While LASSO regularization and nested cross-validation were applied to reduce overfitting risk, they cannot compensate for insufficient event numbers. Larger multicenter cohorts with more CSPCa events are needed to achieve a robust EPV level and further confirm the stability and generalizability of the model. Third, the risk thresholds used for stratification were exploratory probability bands rather than externally validated clinical cutoffs. Fourth, established external risk calculators could not be evaluated because the dataset did not contain all calculator-specific variables. For PI-RADS 3 lesions, a radiomics nomogram incorporating both radiomics signature and PSAD demonstrated that comprehensive evaluation through calibration curves, Hosmer-Lemeshow tests, and decision curve analysis is essential to support individualized biopsy decisions, underscoring that in equivocal populations, calibration and clinical net benefit are critical complements to discrimination metrics (30). Finally, the decision curve analysis was based on internally cross-validated predictions and should be regarded as supportive rather than confirmatory evidence of clinical utility.

In summary, a three-variable logistic regression model based on age, PHI and PHID showed stable internal performance for predicting CSPCa in patients with PI-RADS 3 lesions. The model provides an interpretable framework for risk estimation, but its role in biopsy decision-making should be tested in external cohorts before clinical implementation.

## 5 Conclusion

In this study, we developed and internally validated a parsimonious three-variable logistic model incorporating age, PHI and PHID to predict clinically significant prostate cancer in patients with PI-RADS 3 lesions. In five-fold cross-validation, the model achieved an AUROC of 0.840 with acceptable calibration metrics. We defined exploratory low- and high-risk subgroups based on predicted probabilities, and decision curve analysis demonstrated favorable net benefit over both universal biopsy and no-biopsy strategies across clinically relevant thresholds.

The model delivered significantly better discriminative performance than conventional markers including PSA and PSAD. While its incremental discriminative value over PHI or PHID alone did not reach statistical significance, the combined model yielded lower overall prediction error and improved discriminative performance with acceptable calibration, which support individualized clinical decision-making. As a single-center retrospective study with a limited number of CSPCa events and only internal validation, our estimates may carry modest overoptimism. Further multicenter external validation is warranted to confirm the model’s generalizability and its role in guiding biopsy decisions for PI-RADS 3 patients.

## Data Availability

The raw data supporting the conclusions of this article will be made available by the authors, without undue reservation.

## Supplementary material

Supplementary Figure 1 LASSO logistic regression for feature selection. **(A)** Tuning parameter (λ) selection via 10-fold stratified cross-validation in the full cohort. The x-axis shows log(λ), and the y-axis shows cross-validated log loss. Vertical dashed lines mark lambda.min (red) and lambda.1se (green). **(B)** LASSO coefficient paths of all candidate variables across different values of log(λ). Vertical dashed lines correspond to lambda.min and lambda.1se. age, PHI, and PHID retained non-zero coefficients at both λ values. This full-cohort LASSO fit was performed for descriptive purposes only after completion of the nested internal validation.

Supplementary Figure 2 Decision curve analysis of the final logistic regression model. Analysis is performed based on 5-fold cross-validated out-of-fold predictions from 193 patients with PI-RADS 3 lesions (41 CSPCa events), with fold-wise LASSO feature selection to avoid data leakage. Net benefit is plotted across threshold probabilities ranging from 0.01 to 0.80. The solid gray line represents the “treat all” strategy, and the dotted black line represents the “treat none” strategy. This analysis is considered exploratory in the absence of an external validation cohort.
