# Supplementary material for: Development and internal validation of a parsimonious logistic regression model integrating multiparametric clinical indicators for predicting prostate cancer in the PI-RADS 3 cohort
Source: Front Oncol. 2026 Jul 22;16:1883622. doi: 10.3389/fonc.2026.1883622 (PMC13437321; doi:10.3389/fonc.2026.1883622)
Supplement: Supplementary file 1 [file Table1.docx]

**Table S1. Descriptive full-cohort LASSO feature selection performed after internal validation**

| **Candidate predictor** | **Coefficient at lambda.min** | **Selected at lambda.min** | **Coefficient at lambda.1se** | **Selected at lambda.1se** |
| --- | --- | --- | --- | --- |
| **AGE** | 0.477 | **Yes** | 0.206 | **Yes** |
| **PSA** | 0.000 | No | 0.000 | No |
| **FPSA** | 0.000 | No | 0.000 | No |
| **F/T** | 0.000 | No | 0.000 | No |
| **P2PSA** | 0.000 | No | 0.000 | No |
| **PV** | 0.000 | No | 0.000 | No |
| **PSAD** | 0.000 | No | 0.000 | No |
| **PHI** | 0.672 | **Yes** | 0.591 | **Yes** |
| **PHID** | 0.967 | **Yes** | 0.684 | **Yes** |

This table presents a descriptive LASSO logistic regression fit performed in the full cohort after completion of the primary internal-validation analysis. It is provided to show which candidate predictors retained non-zero coefficients when the final presentation model was summarized in the full dataset, and should not be interpreted as the source of the cross-validated performance estimates. In the primary analysis, feature selection was embedded within each outer training fold of the 5-fold internal-validation workflow. Candidate predictors were standardized before modelling. The tuning parameter was selected by 10-fold stratified cross-validation using cross-validated log loss. Predictors with non-zero coefficients were considered selected. CSPCa, clinically significant prostate cancer; PSAD, prostate-specific antigen density; PHI, Prostate Health Index; PHID, PHI density.
