# Supplementary material for: Development and internal validation of a parsimonious logistic regression model integrating multiparametric clinical indicators for predicting prostate cancer in the PI-RADS 3 cohort
Source: Front Oncol. 2026 Jul 22;16:1883622. doi: 10.3389/fonc.2026.1883622 (PMC13437321; doi:10.3389/fonc.2026.1883622)
Supplement: Supplementary file 2 [file Table2.docx]

**Table S2. Collinearity analysis of predictors in the final logistic regression model**

| **Variable** | **R-squared** | **Tolerance** | **VIF** |
| --- | --- | --- | --- |
| AGE | 0.051 | 0.949 | 1.053 |
| PHI | 0.530 | 0.470 | 2.129 |
| PHID | 0.542 | 0.458 | 2.185 |

Collinearity was assessed for the LASSO-selected predictors included in the final logistic regression model (AGE, PHI, and PHID). Tolerance was calculated as 1/VIF. A VIF value below 5 was considered to indicate no substantial multicollinearity. VIF, variance inflation factor; PHI, Prostate Health Index; PHID, PHI density.
