# Supplementary material for: Development and internal validation of a parsimonious logistic regression model integrating multiparametric clinical indicators for predicting prostate cancer in the PI-RADS 3 cohort
Source: Front Oncol. 2026 Jul 22;16:1883622. doi: 10.3389/fonc.2026.1883622 (PMC13437321; doi:10.3389/fonc.2026.1883622)
Supplement: Supplementary file 3 [file Table3.docx]

**Table S3. Sensitivity, specificity and missed clinically significant prostate cancer count at a predicted-probability threshold of 0.20**

| **Model** | **Threshold** | **Sensitivity (95% CI)** | **Specificity (95% CI)** | **Missed CSPCa, n** | **Missed CSPCa, %** |
| --- | --- | --- | --- | --- | --- |
| Logistic regression | 0.20 | 0.732 (0.581-0.843) | 0.822 (0.754-0.875) | 11 | 26.8 |

CSPCa, clinically significant prostate cancer; CI, confidence interval. At the 0.20 threshold, 136 patients were classified below threshold, among whom 11 had CSPCa. Missed CSPCa (%) was calculated as the proportion of all 41 CSPCa events that fell below the 0.20 threshold.
