# Supplementary material for: Development and internal validation of a parsimonious logistic regression model integrating multiparametric clinical indicators for predicting prostate cancer in the PI-RADS 3 cohort
Source: Front Oncol. 2026 Jul 22;16:1883622. doi: 10.3389/fonc.2026.1883622 (PMC13437321; doi:10.3389/fonc.2026.1883622)
Supplement: Supplementary file 4 [file Table4.docx]

**Table S4. Incremental performance of the final model compared with available clinical predictors**

| **Model or benchmark** | **Predictors** | **AUROC (95% CI)** | **Delta AUROC, final - benchmark (95% CI)** | **P for delta AUROC** | **Brier score** | **Cal. intercept** | **Cal. slope** | **Net benefit at 0.20** | **Net benefit at 0.50** |
| --- | --- | --- | --- | --- | --- | --- | --- | --- | --- |
| Final three-variable model | AGE + PHI + PHID | 0.840 (0.754-0.916) | Reference | - | 0.105 | -0.122 | 0.917 | 0.120 | 0.083 |
| PHID alone | PHID | 0.811 (0.718-0.894) | +0.028 (-0.026 to +0.079) | 0.284 | 0.115 | 0.036 | 1.031 | 0.122 | 0.041 |
| PSAD alone | PSAD | 0.711 (0.621-0.798) | +0.128 (+0.035 to +0.225) | 0.006 | 0.152 | -0.003 | 0.994 | 0.078 | 0.005 |
| PHI alone | PHI | 0.824 (0.739-0.899) | +0.015 (-0.025 to +0.056) | 0.422 | 0.112 | 0.063 | 1.044 | 0.117 | 0.078 |
| PSA alone | PSA | 0.569 (0.463-0.672) | +0.269 (+0.153 to +0.384) | <0.001 | 0.165 | -0.021 | 0.983 | 0.035 | 0.000 |

The final multivariable model is presented as AGE + PHI + PHID, whereas its internal-validation estimates were derived from 5-fold cross-validated out-of-fold predictions with fold-wise LASSO selection. Single-predictor benchmarks were fitted as one-variable logistic models so that AUROC, Brier score and calibration measures were evaluated on the same probability scale. Delta AUROC was calculated as final-model AUROC minus benchmark AUROC; 95% CIs and P values were estimated by paired bootstrap resampling with 1,000 iterations. Net benefit was calculated at threshold probabilities of 0.20 and 0.50. An established external risk calculator was not computed because the required calculator-specific variables were not available in the dataset. AUROC, area under the receiver operating characteristic curve; CSPCa, clinically significant prostate cancer; PHI, Prostate Health Index; PHID, PHI density; PSA, prostate-specific antigen; PSAD, PSA density. Cal., calibration.
